# Probing quantum geometry through optical conductivity and magnetic circular dichroism

**DOI:** 10.1126/sciadv.ado1761

**Published:** 2024-12-18

**Authors:** Barun Ghosh, Yugo Onishi, Su-Yang Xu, Hsin Lin, Liang Fu, Arun Bansil

**Affiliations:** ^1^ Department of Physics, Northeastern University, Boston, MA 02115, USA.; ^2^ Quantum Materials and Sensing Institute, Northeastern University, Burlington, MA 01803, USA.; ^3^ Department of Physics, Massachusetts Institute of Technology, Cambridge, MA 02139, USA.; ^4^ Department of Chemistry and Chemical Biology, Harvard University, MA 02138, USA.; ^5^ Institute of Physics, Academia Sinica, Taipei 11529, Taiwan.

## Abstract

Probing ground-state quantum geometry and topology through optical responses is not only of fundamental interest, but it can also offer several practical advantages. Here, using first-principles calculations on thin films of the antiferromagnetic topological insulator MnBi_2_Te_4_, we demonstrate how the generalized optical weight arising from the absorptive part of the optical conductivity can be used to probe the ground-state quantum geometry and topology. We show that three-septuple-layer MnBi_2_Te_4_ film exhibit an enhanced, almost-perfect magnetic circular dichroism for a narrow photon energy window in the infrared region. We calculate the quantum weight in this MnBi_2_Te_4_ film and show that it far exceeds the lower bound provided by the Chern number. Our results suggest that the well-known optical methods are powerful tools for probing the ground-state quantum geometry and topology.

## Teaser

A light is shone on the topology and quantum geometry of magnetic topological insulators.

## INTRODUCTION

In a crystalline solid, the quantum metric and the Berry curvature together constitute the complex quantum geometry of the Bloch wave functions (*1*). The quantum metric measures the gauge-invariant “distance” between Bloch wave functions at different momenta, whereas the Berry curvature characterizes the change in the phase of the Bloch wave functions along a closed contour in the Brillouin zone (BZ). The quantum geometry of a solid can be directly manifested in its transport properties. The anomalous Hall conductivity can be expressed as an integral of the Berry curvature of the occupied bands over the BZ (*2*, *3*). Recently, it has been shown that the Berry curvature dipole and the quantum metric dipole lead to remarkable nonlinear transport phenomena (*4*–*10*). Effects of quantum geometry on the optical properties of solids are receiving increasing attention (*11*–*17*). The dipole moment matrix element for optical transitions is closely related to the interband Berry connection (*18*). Exploiting this link, a recent study has constructed an alternate description of optical nonlinear responses in the Riemannian geometry notations that involve both ground and excited states (*19*).

The connection between linear optical conductivity and ground-state quantum geometry has remained largely unexplored. Recent theory (*20*) shows that the generalized optical weight, defined as the first negative moment of the absorptive part of optical conductivity (longitudinal and Hall) over the whole frequency range (0<ω<∞) is directly connected to the ground-state quantum geometry and topology. This generalized optical weight is a complex quantity: Its imaginary part, defined by magnetic circular dichroism (MCD), is connected to the ground-state Chern number, whereas its real part, defined by optical absorption, is connected to quantum metric through the Souza-Wilkens-Martin sum rule (*21*). In particular, the real part of the generalized optical weight, recently termed the “quantum weight,” is directly determined by the quantum metric of the occupied band manifold integrated over the BZ. Although the quantum weight is an important ground-state property that quantifies the degree of “quantumness” of an insulating system, it has not been calculated for real materials to our knowledge.

In this work, we use first-principles calculations and effective field theory to study quantum geometry, optical absorption, and MCD in two-dimensional (2D) MnBi_2_Te_4_, a magnetic topological insulator that exhibits trivial and Chern insulator ground states depending on the layer thickness (*22*, *23*). We show by effective field theory that topological band inversion generally increases the quantum weight and thus leads to a strong enhancement of infrared absorption. This is explicitly demonstrated by our first-principles calculations of the frequency-dependent optical conductivity and the generalized optical weight in MnBi_2_Te_4_ films. As the cutoff frequency increases, the generalized weight of MCD converges to the quantized Chern number, whereas the generalized weight of optical absorption converges to the quantum weight, which far exceeds the lower bound provided by the Chern number (*20*). Last, we show that the Chern insulator state in MnBi_2_Te_4_ exhibits an enhanced, almost-perfect MCD for a narrow photon energy window in the infrared region.

## RESULTS

### Optical conductivity and generalized optical weight

For completeness, we first review recent results relating optical conductivity $\sigma_{\alpha \beta }\left(\omega \right)$ to quantum geometry and topology of the ground state (*20*). We evaluate the 2D linear optical conductivity using the Kubo-Greenwood formula for noninteracting electronic systems

$$\sigma_{\alpha \beta }\left(\omega \right)=\frac{e^{2}}{\hbar }\int \left[dk\right]\sum_{n,m}f_{\mathrm{nm}k}\frac{i\epsilon_{\mathrm{mn}k}A_{\mathrm{nm}k}^{\alpha }A_{\mathrm{mn}k}^{\beta }}{\epsilon_{\mathrm{nm}k}+\hbar \omega +\mathrm{i\delta}}$$ (1)

Here, $\epsilon_{nk}$ is the energy eigenvalue of the *n*th Bloch state at crystal momenta k; $\left[dk\right]=d^{2}k/{\left(2\pi \right)}^{2}$ in two dimensions. The interband Berry connection is given by $A_{\mathrm{mn}k}^{\alpha }=\langle u_{mk}|i\partial_{\alpha }|u_{nk}\rangle$, where $|u_{nk}\rangle$ is the cell periodic part of the Bloch wave function. The difference in occupancy, $f_{\mathrm{nm}k}=f_{nk}-f_{mk}$, where $f_{nk}$ is the Fermi distribution function for the *n*th band at k, $\epsilon_{\mathrm{nm}k}=\epsilon_{nk}-\epsilon_{mk}$. The δ is an infinitesimal broadening parameter. Hereafter, we write the frequency-dependent optical conductivity $\sigma_{\alpha \beta }\left(\omega \right)$ as $\sigma_{\alpha \beta }$, omitting the argument ω for the sake of brevity.

The optical conductivity can be divided into symmetric ($\sigma^{L}$) and antisymmetric ($\sigma^{H}$) parts: $\sigma^{L,H}=\left(\sigma_{\alpha \beta }\pm \sigma_{\beta \alpha }\right)/2$ (*24*, *25*). The combination of these two parts together form the absorptive (Hermitian) and reactive (anti-Hermitian) parts: $\sigma^{\mathrm{abs}}=\text{Re}\;\sigma^{L}+i\text{Im}\sigma^{H}$ and $\sigma^{\text{rea}}=\text{Re}\;\sigma^{H}+i\text{Im}\;\sigma^{L}$. Our primary focus is on $\sigma^{\text{abs}}$; its real part ($\text{Re}\;\sigma^{L}$) is related to the absorption of linearly polarized light, whereas its imaginary part ($\text{Im}\;\sigma^{H}$) is responsible for MCD.

We now explore the connection between optical conductivity and ground-state quantum geometry. The quantum geometric tensor for a set of *N* bands parametrized by the wave vector k can be expressed as

$$Q_{\alpha \beta }^{\mathrm{ij}k}=\langle \partial_{\alpha }u_{ik}|\left(1-P_{k}\right)|\partial_{\beta }u_{\mathrm{jk}}\rangle$$ (2)

Here, i,j=1,…,N. The projection operator $P_{k}$ is given by $P_{k}=\sum_{i=1}^{N}|u_{ik}\rangle \langle u_{ik}|$. The real and the imaginary part of $Q_{\mathrm{ij}}^{\alpha \beta }$ represent the non-Abelian quantum metric ($G^{\alpha \beta }$) and the non-Abelian Berry curvature $\left(F^{\alpha \beta }\right)$, i.e., $Q^{\alpha \beta }=G^{\alpha \beta }-\frac{i}{2}F^{\alpha \beta }$. Following (*20*), we define the generalized optical weight using the first negative moment of the absorptive part of the optical conductivity as

$$W_{\alpha \beta }^{1}\left(\omega_{c}\right)=\int_{0}^{\omega_{c}}\mathrm{d\omega}\frac{\sigma_{\alpha \beta }^{\text{abs}}}{\omega }$$ (3)

Here $\omega_{c}$ is a cutoff frequency; in the $\omega_{c}\to \infty$ limit, $W_{\alpha \beta }^{1}$ represents the full spectral weight. For an insulator, $\sigma_{\alpha \beta }^{\text{abs}}$ is nonzero only for photon energy higher than the bandgap; thus, the above integral is convergent. The $\sigma_{\alpha \beta }^{\text{abs}}$ can be recovered from $W^{1}\left(\omega_{c}\right)$ using the formula $\sigma_{\alpha \beta }^{\text{abs}}\left(\omega \right)=\omega {\mathrm{dW}}_{\alpha \beta }^{1}/\mathrm{d\omega}$, and $\sigma_{\alpha \beta }^{\text{rea}}$ can be obtained from $\sigma_{\alpha \beta }^{\text{abs}}$ using the Kramers-Kronig relations. Thus, $W^{1}\left(\omega_{c}\right)$ represents an important quantity that carries all the essential information about the optical conductivity; it represents the integrated dielectric loss below the photon energy $\hbar \omega_{c}$. Following (*20*), we write $W_{\alpha \beta }^{1}\left(\omega_{c}\right)$ as an integral in the k-space

$$\begin{array}{cc} W_{\alpha \beta }^{1}\left(\omega_{c}\right) & =\int_{0}^{\omega_{c}}\mathrm{d\omega}\frac{\sigma_{\alpha \beta }^{\text{abs}}}{\omega }\\ & =\frac{\pi e^{2}}{\hbar }\sum_{o,u}\int_{\epsilon_{\mathrm{uo}}\leq \hbar \omega_{c}}\left[dk\right]A_{\mathrm{ou}}^{\alpha }A_{\mathrm{uo}}^{\beta } \end{array}$$ (4)

For a small cutoff frequency $\omega_{c}$, only a limited region of the k-space and a subset of the bands that satisfies the condition $\epsilon_{\mathrm{uo}}\leq \hbar \omega_{c}$ contribute to the BZ integral. In the $\omega_{c}\to \infty$ limit, the frequency integral represents the full spectral weight when the optical transition involves all the bands, and the k-space integral encompasses the entire BZ.

Hereafter, we will focus on a material that respects the $C_{3z}$ rotation symmetry, which ensures $\sigma^{L}=\sigma_{\mathrm{xx}}=\sigma_{\mathrm{yy}}$ and $\sigma^{H}=\sigma_{\mathrm{xy}}=-\sigma_{\mathrm{yx}}$. Reflecting this assumption, we drop the superscript *L* and *H* and write the symmetric and antisymmetric parts of the optical conductivity as $\sigma_{\mathrm{xx}}$ and $\sigma_{\mathrm{xy}}$, respectively. The relevant real and imaginary part of $W_{\alpha \beta }^{1}$ leads to the identities

$$\int_{0}^{\omega_{c}\to \infty }\mathrm{d\omega}\frac{\text{Re }\sigma_{\mathrm{xx}}}{\omega }=\frac{e^{2}}{2\hbar }K_{\mathrm{xx}}$$ (5)

$$\int_{0}^{\omega_{c}\to \infty }\mathrm{d\omega}\frac{\text{Im}\;\sigma_{\mathrm{xy}}}{\omega }=-\frac{e^{2}}{4\hbar }C_{\mathrm{xy}}$$ (6)

Here, $K_{\mathrm{xx}}$ is the quantum weight, a quantum property of the insulating ground state, given by $K_{\mathrm{xx}}=2\pi \int \left[dk\right]g_{\mathrm{xx}};g_{\mathrm{xx}}=\text{Tr}\left[G_{\mathrm{xx}}\right]$ is the trace of the non-Abelian quantum metric of the occupied band manifold, or alternatively, it represents the Abelian quantum metric of the Slater determinant states formed by the occupied bands. The quantum weight is related to electron localization length in an insulator (*21*), and it represents a quantitative measure of the degree of quantumness in the insulating state (*20*). The Chern number ($C_{\mathrm{xy}}$) in two dimensions is given by the BZ integral of the Berry curvature of the occupied band manifold: $C_{\mathrm{xy}}=2\pi \int \left[dk\right]\sum_{o}F_{\mathrm{xy}}^{o}$. The quantum metric and the Berry curvature of the Slater determinant state of the occupied band manifold must satisfy the inequality (*26*, *27*), $g_{\mathrm{xx}}+g_{\mathrm{yy}}\geq |F_{\mathrm{xy}}|$, which leads to a lower bound on the quantum weight in a 2D system

$$K\equiv K_{\mathrm{xx}}+K_{\mathrm{yy}}\geq |C_{\mathrm{xy}}|$$ (7)

Note that, in the special case of a single occupied band, the quantum geometric tensor is Abelian. Then, if the so-called ideal metric condition $\text{Tr}\;g=|F_{\mathrm{xy}}|$ is satisfied at every k, then the quantum weight equates the lower bound provided by the Chern number: K=∣C∣ (*28*–*30*). Armed with these notations, we now discuss the optical weights and their connection to ground-state quantum geometry and how they lead to enhanced infrared absorption and a near-perfect MCD in a real material.

### Crystal and electronic structure of MnBi_2_Te_4_

**Fig. 1.**
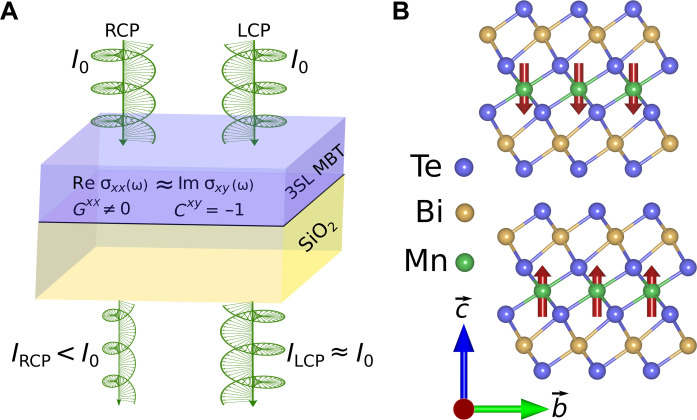
Illustration of MCD and the crystal structure of MnBi_2_Te_4_. (**A**) A schematic illustration of the near-perfect MCD in the 3SL MnBi_2_Te_4_ (MBT) film. $I_{0}$ represents the equal intensity of the RCP and LCP light entering into the medium, whereas $I_{\text{RCP}}\left(I_{\text{LCP}}\right)$ represents the intensity of the RCP (LCP) transmitted beam. When perfect MCD occurs, the circularly polarized light only of a specific helicity is absorbed depending on the sign of $\text{Im}\;\sigma_{\mathrm{xy}}$. (**B**) Crystal structure of 2SL MnBi_2_Te_4_ in the antiferromagnetic phase.

We have chosen to consider MnBi_2_Te_4_, which has recently emerged as the first stoichiometric compound to host an antiferromagnetic topological insulator state (*31*–*38*). In the nonmagnetic phase, it crystallizes in the $R\bar{3}m$ space group. It hosts a layered structure where individual MnBi_2_Te_4_ septuple layers (SLs) are the building blocks in the sequence -Te-Bi-Te-Mn-Te-Bi-Te- atomic layers, which are vertically stacked and stabilized via weak van der Waals forces. Below the magnetic transition temperature, the Mn spins favor an in-plane ferromagnetic coupling (Fig. 1B) and a Neel-type antiferromagnetic ordering in the vertical direction (*39*). Thin films with an odd number of SLs preserve the inversion symmetry (P), whereas an even number of SLs break the inversion and time-reversal symmetries (T) but preserve the combined PT symmetry. In addition, the $C_{3z}$ rotational symmetry is always preserved, irrespective of the number of SLs.

**Fig. 2.**
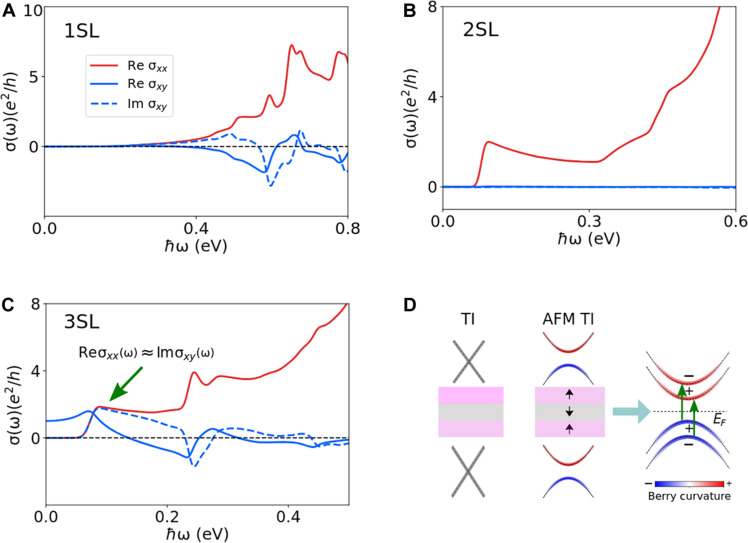
Optical conductivity of a few SL-thick MnBi_2_Te_4_ films. (**A**) 1SL, (**B**) 2SL, and (**C**) 3SL MnBi_2_Te_4_. For 1SL, the $\text{Re}\;\sigma_{\mathrm{xx}}$ increases gradually, whereas for 2SL and 3SL, it has a sudden onset. In the 2SL film, PT symmetry forbids nonzero $\sigma_{\mathrm{xy}}$ at all frequencies. (**D**) A schematic of the low-energy optical transitions involving gapped Dirac cone states of 3SL MnBi_2_Te_4_. The band splitting here arises from the uncompensated magnetization and the hybridization between the top and bottom Dirac cones. The ± sign denotes the parity of the bands. A direct optical transition involving the highest valence band and the lowest conduction band is strongly suppressed due to the optical selection rule.

The low-energy band structure of a few SL MnBi_2_Te_4_ harbors surface-state features of a topological insulator thin film, where two gapless Dirac cones reside on opposite surfaces (*40*). In a few SL MnBi_2_Te_4_ (Fig. 2D), the bandgap at the Dirac crossing is driven by two factors: hybridization between the top and bottom surface Dirac cones and the exchange coupling of the Mn spins. Uncompensated magnetization in the case of an odd number of SLs results in singly degenerate spin-split bands, whereas for the even number of SLs, PT symmetry ensures the double degeneracy of the bands at all crystal momenta. The 1SL MnBi_2_Te_4_ film is topologically trivial, whereas in the 3SL film, the gapped Dirac cone states from the top and bottom surfaces each contribute $e^{2}/2h$ to the Hall conductivity (*31*), leading to a quantum anomalous Hall insulator phase with a Chern number ∣C∣=1 (*22*, *41*, *42*). The even SL MnBi_2_Te_4_ hosts the so-called zero Hall plateau state (*22*, *23*, *37*). Note that the simplified Dirac cone picture used here is an approximation that is valid only near the Fermi level (see the Supplementary Materials for details) (*9*, *36*, *37*, *43*, *44*).

We now proceed to discuss the optical conductivity and the associated optical weights for a few SL-thick MnBi_2_Te_4_ film within the Wannier function–based tight-binding framework (see the Supplementary Materials for details) (*45*). The Hilbert space relevant for the low-frequency response is spanned by bands of Bi p, Te p, and Mn d orbital characters. We consider the low-frequency response first, followed by a discussion the high-frequency optical spectrum.

### Low-frequency response

The optical conductivity of a few SL-thick MnBi_2_Te_4_ film for photon energies in the infrared region is shown in Fig. 2 (A to C). $\sigma^{\text{abs}}$ is directly related to interband transitions so that $\text{Re}\;\sigma_{\mathrm{xx}}$ and $\text{Im}\;\sigma_{\mathrm{xy}}$ both vanish identically for photon energies lower than the bandgap. For the 1SL MnBi_2_Te_4_ film, $\text{Re}\;\sigma_{\mathrm{xx}}$ and $\text{Im}\;\sigma_{\mathrm{xy}}$ increase gradually with frequency, whereas the 2SL and 3SL films exhibit a sudden onset. The $\text{Re}\;\sigma_{\mathrm{xx}}$ displays universal characteristics of optical conductivity in a 2D system, where an interband transition peak is followed by a plateau-like region. Because of the broken T symmetry, 1SL and 3SL films show a finite $\sigma_{\mathrm{xy}}$, whereas in the 2SL film, the combined PT symmetry forbids nonzero $\sigma_{\mathrm{xy}}$ at all frequencies. The 3SL MnBi_2_Te_4_ film hosts the quantum anomalous Hall insulator phase, and in the ℏω→0 limit, $\text{Re}\;\sigma_{\mathrm{xy}}$ starts from the quantized value of $e^{2}/h$ and develops a prominent peak. As indicated by the green arrow in Fig. 1C, the $\text{Re}\;\sigma_{\mathrm{xx}}\approx \text{Im}\;\sigma_{\mathrm{xy}}$ in a narrow frequency window in the 3SL MnBi_2_Te_4_ film. This has important consequences for the MCD, which we will discuss below.

In the 3SL film, the uncompensated magnetization and the hybridization between the top and bottom surface Dirac cones leads to singly degenerate, spin-split bands (Fig. 2D). The low-frequency optical response of a few SL MnBi_2_Te_4_ mostly arises from the interband transitions involving the gapped Dirac cone surface states. Note that a direct optical transition between the highest valence and the lowest conduction band is strongly suppressed due to optical selection rules. Because the system is close to a topological phase transition, and the low-frequency response is driven by the gapped Dirac surface states, we consider the optical conductivity of a gapped Dirac cone model in the next section.

### Enhanced optical absorption due to band inversion

Here, we consider the gapped Dirac cone model as the low-energy effective theory of systems near a topological phase transition. We start with the Dirac Hamiltonian

$$H=\left(-m-k^{2}\right)\sigma_{z}+v\left(k_{x}\sigma_{x}+k_{y}\sigma_{y}\right)$$ (8)

where *m* denotes the mass and *v* is the velocity of the Dirac fermion. In this model, the topological phase transition can be controlled by tuning the mass parameter *m*; the Dirac Hamiltonian describes a topologically trivial (nontrivial) phase when m>0(m<0). The optical absorption of linearly polarized light is described by the real part of the longitudinal conductivity, $\text{Re}\;\sigma_{\mathrm{xx}}$. We obtain an exact analytical expression for $\text{Re}\;\sigma_{\mathrm{xx}}$ for our model as

$$\text{Re}\;\sigma_{\mathrm{xx}}\left(\omega \right)=\frac{e^{2}}{4\hbar \Omega^{2}}\sum_{i}\frac{2M^{2}+2q_{i}^{4}+q_{i}^{2}}{|2M+2q_{i}^{2}+1|}$$ (9)

where $M=m/v^{2},\Omega =\hbar \omega /v^{2}$ are the renormalized mass and frequency, and $\epsilon_{\pm }=\pm \sqrt{{\left(m+k^{2}\right)}^{2}+v^{2}k^{2}}$ is the energy dispersion of the Dirac fermion. The $q_{i}=k_{i}/v$ is a renormalized wave vector at which the resonance occurs and is a function of the frequency. An explicit form of $q_{i}$ is provided in the Supplementary Materials.

**Fig. 3.**
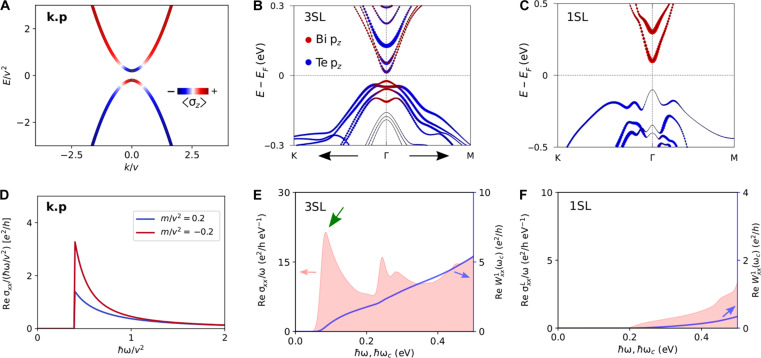
Enhanced infrared absorption due to topological band inversion. (**A**) Band inversion in the k.p model; colors denote spin polarization. (**B** and **C**) Orbital-resolved band structure of a (B) 3SL and (C) 1SL MnBi_2_Te_4_ film. The 3SL film displays band inversion between the Bi $p_{z}$ and Te $p_{z}$ orbitals near the Γ point, which is absent in the 1SL film. (**D**) $\text{Re}\;\sigma_{\mathrm{xx}}/\omega$ calculated from the k.p model in the topological and nontopological phase. (**E** and **F**) $\text{Re}\;\sigma_{\mathrm{xx}}/\omega$ and the corresponding optical weight $\text{Re}\;W_{\mathrm{xx}}^{1}$ for the (E) 3SL and (F) 1SL film. A clear enhancement of $\text{Re}\;\sigma_{\mathrm{xx}}/\omega$ in the topological phase (m<0) compared to the trivial phase (m>0) is seen for the same magnitude of the gap (2∣m∣). This is further supported by our first-principles results: For the 1SL case, $\text{Re}\;\sigma_{\mathrm{xx}}/\omega$ increases gradually, whereas for the 3SL film, it has a sharp peak in the low-frequency region.

As shown in Fig. 3D, the topological phase (m<0) clearly exhibits a larger optical response than the trivial phase for the same value of the gap (2∣m∣). This can be seen more clearly from Eq. 9. For simplicity, we consider the response at the band edge where $m\leq v^{2}/2$. At frequency $\omega_{0}=2m/\hbar$, the optical transition occurs at *k* = 0, and hence $\text{Re}\;\sigma_{\mathrm{xx}}\left(\omega_{0}\right)\propto {|v^{2}+2m|}^{-1}$. Therefore, the optical conductivity at the band edge is always larger for m<0 than for m>0 for the same value of ∣m∣. This indicates that the band inversion enhances the optical response near the topological phase transition.

This result is well supported by our first-principles calculations on a few SL MnBi_2_Te_4_ film. Figure 3 (B and C) shows that the 3SL film hosts a clear band inversion between the Bi $p_{z}$ and Te $p_{z}$ orbital–derived states near the Γ point, whereas this band inversion is absent in the 1SL film due to quantum confinement effects. In the 1SL film, $\text{Re}\;\sigma_{\mathrm{xx}}/\omega$ increases gradually with frequency, whereas the 3SL film shows a sudden onset in the low-frequency region, indicating an enhanced infrared absorption and a larger quantum weight.

Band inversion–induced enhancement in optical absorption can be understood from the presence of a large quantum metric carried by the low-energy bands of the 3SL MnBi_2_Te_4_ film. Here, the wave function of the low-energy bands near the Γ point changes rapidly between two nearby k points, resulting in a large quantum metric in this region. $\text{Re}\;\sigma_{\mathrm{xx}}/\omega$ is directly connected to the quantum metric, and band inversion thus generally leads to enhanced optical absorption.

### Chern number as the weight of MCD

**Fig. 4.**
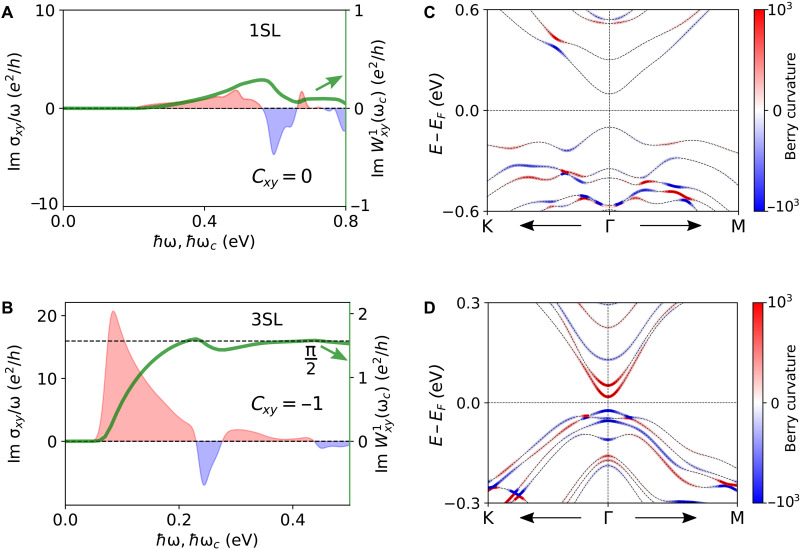
Imaginary part of the optical weight and its connection to the Chern number. (**A** and **B**) $\text{Im}\;\sigma_{\mathrm{xy}}/\omega$ $\left(\text{Im}\;W_{\mathrm{xy}}^{1}\right)$ varying photon energy (cutoff energy) for the (A) 1SL and (B) 3SL MnBi_2_Te_4_ film. In the case of 1SL, the $\text{Im}\;W_{\mathrm{xy}}^{1}$ converges to zero, whereas for the 3SL case, $\text{Im}\;W_{\mathrm{xy}}^{1}$ converges to $e^{2}/4\hbar$, revealing their trivial and Chern insulator ground state, respectively. (**C** and **D**) Band-resolved and momentum-resolved Berry curvature distribution for the (C) 1SL and (D) 3SL MnBi_2_Te_4_ film. In the 3SL case, the low-energy bands carry a large Berry curvature around a small region near the Γ point. The broadening parameter was set to 10 meV for (A) and (B).

Turning to the optical weight arising from $\text{Im}\;\sigma_{\mathrm{xy}}$, which is responsible for the MCD, we see from Fig. 4 (A and B) that, in the 1SL film, $\text{Im}\;\sigma_{\mathrm{xy}}/\omega$ increases gradually with the photon energy and shows an oscillatory behavior. In contrast, for the 3SL film, $\text{Im}\;\sigma_{\mathrm{xy}}/\omega$ has a large peak at ℏω≈85 meV, and it displays a diminishing oscillatory pattern with increasing frequency. The corresponding optical weight, $\text{Im}\;W_{\mathrm{xy}}^{1}$, for the 1SL film, starts from zero and gradually increases with the cutoff frequency before approaching the horizontal axis, indicating the trivial nature of the 1SL the film. In contrast, for the 3SL film, $\text{Im}\;W_{\mathrm{xy}}^{1}$ starts from zero and quickly approaches the quantized value of $e^{2}/4\hbar$, revealing the Chern insulator ground state ($C_{\mathrm{xy}}=-1$) of the 3SL film. The weight arising from the first peak of $\text{Im}\;\sigma_{\mathrm{xy}}/\omega$ is sufficient to saturate the quantized Chern number of the 3SL film.

The rapid convergence of $\text{Im}\;W_{\mathrm{xy}}^{1}$ with cutoff frequency for the 3SL film can be understood from the Berry curvature distribution in the k-space. As already pointed out above, the low-energy bands of the 3SL film are the gapped Dirac cone surface states of a magnetic topological insulator thin film and carry a large Berry curvature that is highly concentrated in a small k-space region (Fig. 4D) near the Γ point. The optical transitions at low frequency mostly involve these gapped Dirac-like bands. Therefore, following Eq. 4, for a cutoff energy of $\hbar \omega_{c}\approx 23$ meV, the low-energy bands around a small k-space region near the Γ point that satisfies $\hbar \omega_{c}\leq \epsilon_{\mathrm{uo}k}$ are sufficient to saturate the quantized Chern number of the 3SL film.

### Optical rotations and perfect MCD

We quantify now the MCD arising from $\text{Im}\;\sigma_{\mathrm{xy}}$ and show that the 3SL MnBi_2_Te_4_ film in the Chern insulator phase exhibits an enhanced near-perfect MCD for a narrow photon energy window in the infrared region. For completeness, we also quantify the complex Faraday and Kerr rotation angles that arise from a nonzero $\sigma_{\mathrm{xy}}$. The real part, θ, of the complex Kerr (Faraday) angle is representative of the rotation in the plane of linearly polarized incident light after reflection (transmission), and it is directly related to $\text{Re}\;\sigma_{\mathrm{xy}}$; the imaginary part, η, represents the ellipticity of the reflected (transmitted) beam, and it arises from $\text{Im}\;\sigma_{\mathrm{xy}}$, which, in turn, lead to circular dichroism (CD). Using the frequency-dependent rotation angles and the MCD, the $\sigma_{\mathrm{xy}}$ and $\sigma_{\mathrm{xx}}$ can be obtained.

We consider a realistic experimental setup, where the MnBi_2_Te_4_ thin film on a SiO_2_ substrate is placed in vacuum (Fig. 1A). For normal incidence of linearly polarized light, the complex Faraday (${\tilde{\Theta }}_{F}$) and Kerr (${\tilde{\Theta }}_{K}$) angles in the thin film limit can be approximated as (*46*–*49*)

$${\tilde{\Theta }}_{F}=\theta_{F}+i\eta_{F}=\frac{\mu_{0}c\sigma_{\mathrm{xy}}}{1+n_{\text{sub}}+\mu_{0}c\sigma_{\mathrm{xx}}}$$ (10)

$${\tilde{\Theta }}_{K}=\theta_{K}+i\eta_{K}=\frac{2\mu_{0}c\sigma_{\mathrm{xy}}}{1-{\left(n_{\text{sub}}+\mu_{0}c\sigma_{\mathrm{xx}}\right)}^{2}}$$ (11)

**Fig. 5.**
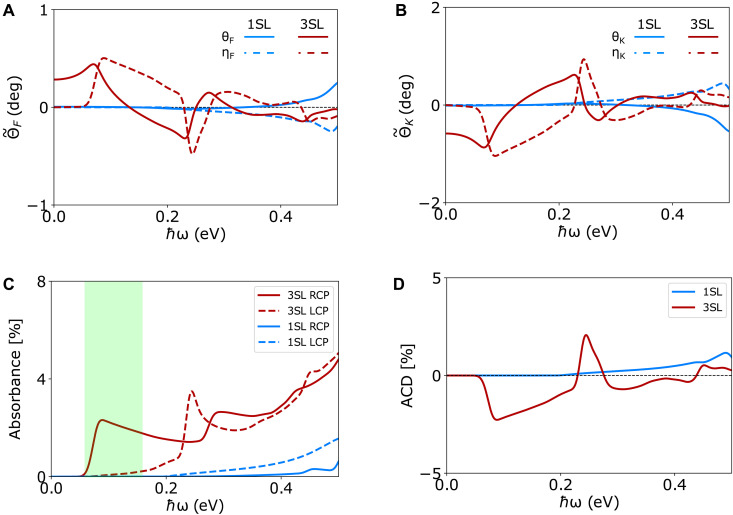
Optical rotations and near-perfect MCD. Complex (**A**) Faraday angles and (**B**) Kerr angles for the 1SL and 3SL MnBi_2_Te_4_ film. deg, degrees. (**C**) Absorption probability of circularly polarized light of different helicities for the 1SL and 3SL films. The 3SL film exhibits an enhanced almost-perfect MCD for 65≲ℏω≲150 meV. (**D**) Resulting absorptive CD.

Here, $\sigma_{\mathrm{xy}}$ and $\sigma_{\mathrm{xx}}$ are the 2D conductivities in reciprocal ohm unit. $n_{\text{sub}}$ is the refractive index of the substrate, and the incident medium is the vacuum. Using $n_{\text{sub}}=\sqrt{\epsilon_{\mathrm{xx}}^{{\text{SiO}}_{2}}}=1.97$, in the limiting case, we obtain $\theta_{F}\propto \text{Re}\;\sigma_{\mathrm{xy}}$ and $\eta_{F}\propto \text{Im}\;\sigma_{\mathrm{xy}}$. As shown in Fig. 5A, the photon energy dependence of $\theta_{F}$ and $\eta_{F}$ largely follows the $\text{Re}\;\sigma_{\mathrm{xy}}$ and $\text{Im}\;\sigma_{\mathrm{xy}}$, respectively. In the ℏω→0 limit, for the 1SL case, $\theta_{F}$ vanishes due to its trivial nature, whereas for the 3SL film, $\theta_{F}$ has a finite value (≈0.28°), which depends on the substrate refractive index, and in the case of a freestanding 3SL film, in the ℏω→0 limit, $\theta_{F}$ attains the universal quantized value of $\theta_{F}=\left(\alpha_{\text{fine}}\right)\approx 0.42{}^{\circ}$; here $\alpha_{\text{fine}}\left(\approx 1/137\right)$ is the fine-structure constant (*43*, *50*–*52*). The complex Kerr angle shares a similar pattern as the Faraday angle, although it has an opposite relative sign. Depending on the substrate refractive index and the choice of the broadening parameter (δ), the $\theta_{K}$ can reach values ∼1°, comparable to other magnetic 2D materials (*53*). As evident from the $\eta_{K}$ and $\eta_{F}$ results, the transmitted and the reflected light both become elliptically polarized. The larger magnitude of $\eta_{K}$ in comparison to $\eta_{F}$ suggests that the reflected light attains a higher degree of ellipticity than the transmitted beam. The nonzero η results in MCD.

Absorption probability for circularly polarized light of helicity $\hat{\chi }$ [$\hat{\chi }=+1$ for left circularly polarized (LCP) and $\hat{\chi }=-1$ for right circularly polarized (RCP)] for MnBi_2_Te_4_ thin films on a substrate can be calculated using

$$A_{\hat{\chi }}=\frac{4\mu_{0}c\left(\text{Re}\;\sigma_{\mathrm{xx}}-\hat{\chi }\text{Im}\;\sigma_{\mathrm{xy}}\right)}{{|1+n_{\text{sub}}+\mu_{0}c\left(\sigma_{\mathrm{xx}}+i\hat{\chi }\sigma_{\mathrm{xy}}\right)|}^{2}}$$ (12)

Figure 5C shows that circularly polarized light of opposite helicity is absorbed differently in the few SL MnBi_2_Te_4_ films due to the presence of a finite $\sigma_{\mathrm{xy}}$. The resulting absorptive CD ($\mathrm{ACD}=A_{\text{LCP}}-A_{\text{RCP}}$) is quantified in Fig. 5D. In the 1SL case, we observe a gradual increase in the absorbance for photon energies higher than the bandgap. In contrast, an almost-perfect MCD is observed for the 3SL MnBi_2_Te_4_ film in the photon energy range 65≲ℏω≲150 meV. Note that, in this photon energy window, $\text{Re}\;\sigma_{\mathrm{xx}}$ is nearly equal to $\text{Im}\;\sigma_{\mathrm{xy}}$ so that only the RCP light is absorbed, whereas the absorption of the LCP light almost vanishes. Although at ℏω≈85 meV, the absorption probability for RCP reaches its maximum value, it is still only ~2.3% due to the 2D nature of the few-layered MnBi_2_Te_4_. This value could, however, be enhanced by adjusting the refractive indices of the incident medium and the substrate. The 3SL film with the opposite magnetic configuration (↑↓↑ versus ↓↑↓) only absorbs the LCP light instead of the RCP light. The LCP and RCP lights are absorbed almost equally at higher photon energies, resulting in a small CD.

The nearly perfect MCD in the 3SL film can be understood from the absence of dissipation through Joule heating in a 2D material for incident light of a specific helicity. For circularly polarized light of helicity $\hat{\chi }$, the power dissipation through Joule heating in a 2D conductor can be estimated as

$$P_{\hat{\chi }}=\text{Re}\left(j^{*}.E\right)\propto \text{Re}\;\left(\sigma_{\mathrm{xx}}+\sigma_{\mathrm{yy}}\right)-2\hat{\chi }\text{Im}\;\sigma_{\mathrm{xy}}$$ (13)

Here, j is the surface current density induced by the electric field E of the circularly polarized incident light. Clearly, in the $C_{3z}$ symmetric 3SL film, the $P_{\hat{\chi }}$ vanishes for circularly polarized light of helicity $\hat{\chi }$ when $\text{Re}\;\sigma_{\mathrm{xx}}=\hat{\chi }\text{Im}\;\sigma_{\mathrm{xy}}$, resulting in the absorption of circularly polarized light of opposite helicity $-\hat{\chi }$. In terms of the quantum geometry, for a two-band system with a single occupied band, this requires the equality $g_{\mathrm{xx}}\left(k\right)=F_{\mathrm{xy}}\left(k\right)/2$ for wave vector k at which the optical transition occurs. Note that this condition holds for a two-bandgapped Dirac system at *k* = 0

$$g_{\mathrm{xx}}\left(k=0\right)=|F_{\mathrm{xy}}\left(k=0\right)|/2$$ (14)

Figure 2D shows that the low-energy optical excitation of 3SL MnBi_2_Te_4_ only involves two bands, each originating from the Dirac cone surface states. In particular, the transitions occurring around ℏω≈85 meV are at k=0 where Eq. 14 is satisfied; hence ≈100% MCD is observed. At higher frequencies, the optical transitions occur away from k=0, and the ≈100% MCD is no longer realized. Nevertheless, because of the unique electronic structure of the 3SL film, the absorption probability of the LCP is still an order of magnitude lower than the RCP up to ℏω≈150 meV. We expect our observation of an enhanced perfect MCD to be valid in thicker MnBi_2_Te_4_ films with an odd number of SLs (5SL, 7SL, etc.; see the Supplementary Materials), where the Chern insulator phase has been experimentally observed (*41*). To the best of our knowledge, such an enhanced almost-perfect MCD in a reasonably wide photon energy window (65≲ℏω≲150 meV) in the infrared region has not been reported so far for a single-phase solid-state material. Although our computations correctly capture interband transitions, excitonic effects are not accounted for, and in principle, a perfect MCD may be possible for photon energies below the bandgap.

### High-frequency response and the quantum weight

**Fig. 6.**
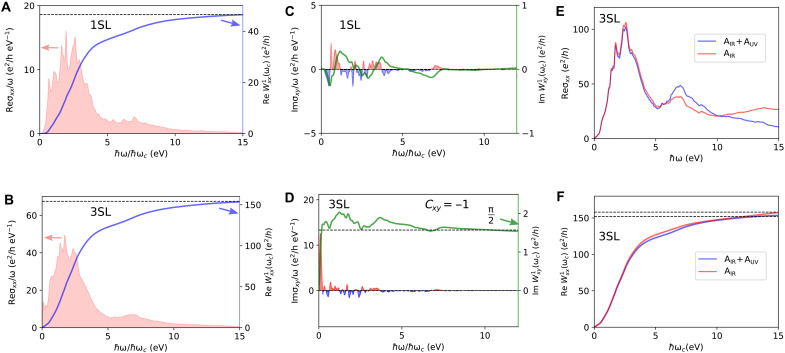
Optical conductivity and optical weight of a few SL-thick MnBi_2_Te_4_ films over a wide photon energy window. (**A** and **B**) $\text{Re}\;\sigma_{\mathrm{xx}}/\omega$ ($\text{Re}\;W_{\mathrm{xx}}^{1}$) for the (A) 1SL and (B) 3SL film. $\text{Re}\;W_{\mathrm{xx}}^{1}$ steadily converges to the quantum weight, the quantum metric of the occupied band manifold integrated over the BZ. (**C** and **D**) $\text{Im}\;\sigma_{\mathrm{xy}}/\omega$ $\left(\text{Im}\;W_{\mathrm{xy}}^{1}\right)$ for the (C) 1SL and (D) 3SL film. In the 1SL case, $\text{Im}\;W_{\mathrm{xy}}^{1}$ converges to zero, whereas for the 3SL film, $\text{Im}\;W_{\mathrm{xy}}^{1}$ converges to $e^{2}/4\hbar$, revealing their trivial and nontrivial ground states, respectively. (**E** and **F**) Effect of ignoring the UV part ($A^{\text{UV}}$) of the interband Berry connection on (E) $\text{Re}\;\sigma_{\mathrm{xx}}$ and (F) $\text{Re }W_{\mathrm{xx}}^{1}$. The broadening parameter was set to 50 meV.

Turning to the high-frequency optical response of a few SL-thick MnBi_2_Te_4_ films, we consider $\sigma_{\alpha \beta }^{\text{abs}}$ and calculate the optical weight $W_{\alpha \beta }^{1}$ by varying the cutoff frequency over a wide frequency range. The high-frequency behavior of $\text{Re}\;\sigma_{\mathrm{xx}}$ is presented in the Supplementary Materials. $\text{Re}\;\sigma_{\mathrm{xx}}$ at high frequencies overall displays similar characteristics irrespective of the number of layers: It steadily increases with the frequency and reaches its maximum value when the number of available optical transition channels is maximum, before it starts to decrease. As shown in Fig. 6 (A and B), for the 1SL and 3SL films at high frequencies, $\text{Re}\;\sigma_{\mathrm{xx}}/\omega$ largely follows $\text{Re}\;\sigma_{\mathrm{xx}}$ in that it increases steadily, reaches the peak value, and then decreases. We evaluate the optical weight $\text{Re}\;W_{\mathrm{xx}}^{1}$ following Eq. 3 by varying the cutoff frequency. As evident from Fig. 6 (C and D), the $\text{Re}\;W_{\mathrm{xx}}^{1}$ steadily increases with the cutoff frequency and converges for $\hbar \omega_{c}≳12$ eV. From this converged value of $\text{Re}\;W_{\mathrm{xx}}^{1}$, we deduce the quantum weight $K_{\mathrm{xx}}$ using Eq. 5. The total single-particle quantum weight *K* is given by $K=2K_{\mathrm{xx}}$ due to the $C_{3z}$ rotation symmetry. For the 1SL and 3SL films, we obtain the total quantum weight $K^{1\text{SL}}=29.75$ and $K^{3\text{SL}}=98.45$, respectively. Although the $\sigma_{\mathrm{xy}}$ vanishes identically at all frequencies, $\text{Re}\;\sigma_{\mathrm{xx}}$ leads to a nonzero quantum weight for the 2SL film (see the Supplementary Materials). Although the quantum weight far exceeds the Chern number in a material such as 3SL MnBi_2_Te_4_, as pointed out in (*20*), the K≥C bound holds fairly well in twisted bilayer MoTe_2_ at θ=1.7° twist angle and, for twisted bilayer WTe_2_, at θ=1.7° twist angle, where K=1.07 and K=1.04, respectively, with ∣C∣=1. Notably, the current formalism for computing quantum weights depends on the number of unoccupied bands, although due to the 1/ω factor, we expect contributions from higher energy states to be small. In this connection, it would be interesting to compute the quantum weight using recently proposed methods (*54*).

Considering the imaginary part of $\sigma_{\alpha \beta }^{\text{abs}}$, $\text{Im}\;\sigma_{\mathrm{xy}}$ has a bounded oscillatory pattern that can take both negative and positive values (see the Supplementary Materials). Figure 6 (C and D) shows that, for the 1SL film, $\text{Im}\;\sigma_{\mathrm{xy}}/\omega$ shows a gradual increase in the low-frequency region and an oscillatory behavior at high frequencies. In contrast, for the 3SL film, $\text{Im}\;\sigma_{\mathrm{xy}}/\omega$ has a large peak at low frequencies and a diminishing oscillatory pattern at high frequencies. The corresponding optical weight, $\text{Im}\;W_{\mathrm{xy}}^{1}$, for the 1SL film starts from zero and oscillates around the horizontal axis before converging around $\hbar \omega_{c}\approx 7$ eV. In contrast, in in the 3SL film, $\text{Im}\;W_{\mathrm{xy}}^{1}$ starts from zero, quickly approaches the quantized value of $e^{2}/4\hbar$, and oscillates around $y=e^{2}/4\hbar$, reflecting its Chern insulator ground state ($C_{\mathrm{xy}}=-1$). It is clear that $\text{Im}\;W_{\mathrm{xy}}^{1}$ can differentiate between the trivial and nontrivial insulating ground states.

$\text{Re}\;\sigma_{\mathrm{xx}}$ is related to the optical absorption and quantum metric, and it is always positive, resulting in a slower convergence of $\text{Re}\;W_{\mathrm{xx}}^{1}$ with the cutoff frequency. On the other hand, $\text{Im}\;\sigma_{\mathrm{xy}}$ is related to the MCD and Berry curvature, and it can be both positive and negative. The Berry curvature effect dominates in the low-frequency region when the optical transitions mostly involve topological bands that carry large Berry curvatures. Consequently, $\text{Im}\;W_{\mathrm{xy}}^{1}$ converges rapidly within a small cutoff frequency range, and the converged quantum weight far exceeds the lower bound provided by the quantized Chern number in a material.

It should be noted that, when computing the optical response through Peierls substitution using a tight-binding model, part of the contribution arising from the off-diagonal position matrix elements of the atomic orbitals is generally not captured. In a tight-binding model, electrons are assumed to be bound to the atomic sites, and the optical response arises only from the hopping of the electrons. In such a scenario, if the hopping of the tight-binding model is completely turned off, then the optical response vanishes. However, in real systems, the optical response can be finite even for an isolated array of atoms without any hopping, where the optical response can be driven by transitions between different atomic orbitals of the same atom. This contribution becomes important at high frequencies. To account for this effect, the total interband Berry connection can be split into two parts: $A=A^{\text{IR}}+A^{\text{UV}}$ (see the Supplementary Materials for details). $A^{\text{IR}}$ arises from the hopping of electrons, whereas $A^{\text{UV}}$ is related to the optical excitations involving orbitals of individual atoms. We highlight the effect of ignoring the $A^{\text{UV}}$ term on the $\text{Re}\;\sigma_{\mathrm{xx}}$ and the resulting quantum weight in Fig. 6 (E and F). It is clear from these results that the $A^{\text{UV}}$ part has a non-negligible contribution to $\text{Re}\;\sigma_{\mathrm{xx}}$ at the high frequencies, and therefore it can influence the converged value of the quantum weight (see the Supplementary Materials). The $A^{\text{UV}}$ part does not have a substantial contribution to $\text{Im}\;\sigma_{\mathrm{xy}}$, or equivalently, to the Chern number (see the Supplementary Materials). The nonzero Chern number arises from the low-energy topological bands, and therefore, the $A^{\text{IR}}$ contribution is sufficient to saturate the quantized Chern number. Note that the $A^{\text{UV}}$ contribution also depends on the spread of the Wannier function, which can be optimized by performing maximum localization procedures, and this contribution is not unambiguous. Therefore, it is important to include the ultraviolet (UV) contribution for an unambiguous determination of the quantum weight.

## DISCUSSION

To summarize, using first-principles calculations and effective field theory, we demonstrate, based on a case study of a real material, how the absorptive part of the optical conductivity is connected to the ground-state quantum geometry and topology. In a quantum anomalous Hall insulator, where the Berry curvature is often highly concentrated around a small region in the momentum space, the optical weight of the MCD within a narrow frequency range can be sufficient to saturate the quantized Chern number. We show that the 3SL MnBi_2_Te_4_ film in the Chern insulator state exhibits enhanced, almost-perfect MCD in a narrow photon energy range in the infrared region. We quantify the quantum weight and show that it far exceeds the lower bound provided by the Chern number. Our results connect the optical response to ground-state quantum geometry and topology in a real material to provide previously unidentified design strategies for next-generation optoelectronic devices by exploiting the quantum geometric aspects of the topological states.

## MATERIALS AND METHODS

### Computational details

We used the Vienna ab initio simulation package to perform density functional theory–based first-principles calculations (*55*). The kinetic energy cutoff for the plane-wave basis was set to 400 eV (*56*). We used a Γ-centered 8×8×1 k-grid to perform the BZ integral and adopted the GGA (Generalized Gradient Approximation)+*U* approach and set *U* = 3.0 eV to treat the localized Mn 3d orbitals (*57*). The Wannier function–based tight-binding model was constructed using the Wannier90 suite of codes (*58*). The optical conductivity was calculated using a home-built code with 1001×1001 k-grid for the BZ integration. Experimental lattice parameters were used.

We compute the frequency-dependent optical conductivity for a few SL-thick MnBi_2_Te_4_ using the Wannier function–based tight-binding framework. Within this formalism, a set of N Bloch bands can be mapped to a set of *N* Wannier functions per unit cell, which serves as the effective basis (*24*, *58*–*61*). The momentum space Hamiltonian in the Wannier basis is given by

$$H_{\mathrm{mn}k}^{\left(W\right)}=\sum_{R}e^{ik\cdot \left(R+\tau_{n}-\tau_{m}\right)}\langle 0m|\hat{H}|Rn\rangle$$ (15)

The $\tau_{n}=\langle 0n|\hat{r}|0n\rangle$ is the center of the *n*th Wannier orbital in the home unit cell. This momentum space Hamiltonian is diagonalized to obtain the U matrix

$$H^{\left(B\right)}{\left(k\right)}_{\mathrm{nm}}={\left(U^{†}\left(k\right)H^{\left(W\right)}\left(k\right)U\left(k\right)\right)}_{\mathrm{nm}}=\epsilon_{n}\left(k\right)\delta_{\mathrm{nm}}$$ (16)

The superscripts “W” and “B” denote the Wannier and the Bloch basis, respectively. The interband (n≠m) Berry connection in the Bloch basis can be split into two parts

$$A=A^{\text{IR}}+A^{\text{UV}}$$ (17)

Here

$$A_{\mathrm{nm}}^{\text{IR}}=i{\left(U^{†}\nabla U\right)}_{\mathrm{nm}}=-\frac{i{\left(U^{†}v^{\left(W\right)}U\right)}_{\mathrm{nm}}}{\epsilon_{\mathrm{nm}}}$$ (18)

where $v^{\left(W\right)}$ is the velocity matrix on the Wannier basis. The UV contribution

$$A^{\text{UV}}=U^{†}A^{W}U$$ (19)

where the $A^{W}$ in terms of the position matrix element in the Wannier basis is given by

$$A_{\mathrm{mn}}^{\left(W\right)}=\sum_{R}e^{ik\cdot \left(R+\tau_{n}-\tau_{m}\right)}\langle 0m|\hat{r}-\tau_{n}|Rn\rangle$$ (20)

The second term, in Eq. 17, vanishes if the position operator is diagonal in the Wannier basis. In practice, these terms are nonvanishing, and ignoring them yields results in the so-called “diagonal tight-binding approximation” (*58*–*60*). Its effect on the $\sigma^{\text{abs}}$ and the quantum weight are shown in the main text.

We evaluate the 2D linear optical conductivity using the Kubo-Greenwood formula for the noninteracting electronic systems as given by

$$\sigma_{\alpha \beta }\left(\hbar \omega \right)=\frac{e^{2}}{\hbar }\int \left[dk\right]\sum_{n,m}f_{\mathrm{nm}k}\frac{i\epsilon_{\mathrm{mn}k}A_{\mathrm{nm}k}^{\alpha }A_{\mathrm{mn}k}^{\beta }}{\epsilon_{\mathrm{nm}k}+\hbar \omega +\mathrm{i\delta}}$$ (21)

The $\sigma_{\alpha \beta }$ can be divided into the absorptive (Hermitian) and the reactive (anti-Hermitian) parts

$$\sigma_{\alpha \beta }^{\text{abs}}=\frac{\pi e^{2}}{\hbar }\int \left[dk\right]\sum_{n,m}f_{\mathrm{nm}k}\epsilon_{\mathrm{mn}k}A_{\mathrm{nm}k}^{\alpha }A_{\mathrm{mn}k}^{\beta }\delta \left(\epsilon_{\mathrm{mn}k}-\hbar \omega \right)$$ (22)

$$\sigma_{\alpha \beta }^{\text{rea}}=\frac{{\mathrm{ie}}^{2}}{\hbar }\int \left[dk\right]\sum_{n,m}f_{\mathrm{mn}k}\text{Re}\left[\frac{\epsilon_{\mathrm{mn}k}}{\epsilon_{\mathrm{mn}k}-\left(\hbar \omega +\mathrm{i\delta}\right)}\right]A_{\mathrm{nm}k}^{\alpha }A_{\mathrm{mn}k}^{\beta }$$ (23)

The symmetric and antisymmetric parts can be calculated using these reactive and absorptive parts. We adopted a Gaussian broadening to approximate the Dirac delta function.
