# Supplementary material for: Probing quantum geometry through optical conductivity and magnetic circular dichroism
Source: Sci Adv. 2024 Dec 18;10(51):eado1761. doi: 10.1126/sciadv.ado1761 (PMC13108739; doi:10.1126/sciadv.ado1761)
Supplement: Supplementary file 1 — Supplementary Text Figs. S1 to S7 [file sciadv.ado1761_sm.pdf]

Science Advances
AAAS

Supplementary Materials for

Probing quantum geometry through optical conductivity and magnetic circular dichroism

Barun Ghosh *et al.*

Corresponding author: Barun Ghosh, b.ghosh@northeastern.edu; Liang Fu, liangfu@mit.edu; Arun Bansil, ar.bansil@northeastern.edu

Sci. Adv. **10**, eado1761 (2024)
DOI: 10.1126/sciadv.ado1761

This PDF file includes:

Supplementary Text
Figs. S1 to S7

Analytical results for the optical absorption in gapped Dirac model

Here, we provide details on calculating the optical response of the Dirac systems. As we explained in the main text, the optical absorption is described by the real part of the longitudinal conductivity, Re $\sigma_{xx}(\omega)$, and the imaginary part of the Hall conductivity, Im $\sigma_{xy}(\omega)$. For the Dirac Hamiltonian in the main text, $H = (-m - k^2)\sigma_z + v(k_x\sigma_x + k_y\sigma_y)$, they are given by

$$
\begin{aligned}
\text{Re}\,\sigma_{xx}(\omega) &= \frac{\omega e^2}{16v^2}\int_0^\infty k\mathrm{d}k\ \delta({}_+(\vec{k}) -{}_-(\vec{k}) - \hbar\omega) \\
&\quad\times \frac{2M^2 + 2(k/v)^4 + (k/v)^2}{(M + (k/v)^2)^2 + (k/v)^2} \\
&= \frac{e^2}{4\hbar\Omega^2}\sum_i \frac{2M^2 + 2q_i^4 + q_i^2}{\left|2M + 2q_i^2 + 1\right|}
\end{aligned}
\tag{S1}
$$

$$
\begin{aligned}
\text{Im}\,\sigma_{xy}(\omega) &= -\frac{\omega e^2}{8v^2}\int_0^\infty k\mathrm{d}k\ \delta({}_+(\vec{k}) -{}_-(\vec{k}) - \hbar\omega) \\
&\quad\times \frac{-M + (k/v)^2}{\left((M + (k/v)^2)^2 + (k/v)^2\right)^{3/2}} \\
&= -\frac{e^2}{4\hbar\Omega}\sum_i \frac{-M + q_i^2}{\left|2M + 2q_i^2 + 1\right|}
\end{aligned}
\tag{S2}
$$

where $M = m/v^2$, $\Omega = \hbar\omega/v^2$ are the renormalized mass and frequency, and ${}_\pm = \pm\sqrt{(m + k^2)^2 + v^2k^2}$ is the energy dispersion of the Dirac fermion. $q_i = k_i(\omega)/v$ is a renormalized wavevector at which the resonance occurs and a function of frequency. q_i is given for $M \geq -1/2$ as

$$
q_i = \begin{cases} \text{(No solution)} & \text{if } (\Omega/2)^2 < M^2 \\ \sqrt{-M - \frac{1}{2} + \sqrt{\left(\frac{\Omega}{2}\right)^2 + M + \frac{1}{4}}}. & \text{if } (\Omega/2)^2 \geq M^2 \end{cases}
\tag{S3}
$$

and for $M \leq -1/2$,

$$
q_i = \begin{cases} \text{(No solution)} & \text{if } \Omega^2 < -4M - 1 \\ \sqrt{-M - \frac{1}{2} + \sqrt{\left(\frac{\Omega}{2}\right)^2 + M + \frac{1}{4}}} & \text{if } \Omega^2 = -4M - 1 \\ \sqrt{-M - \frac{1}{2} \pm \sqrt{\left(\frac{\Omega}{2}\right)^2 + M + \frac{1}{4}}} & \text{if } -4M - 1 < \Omega^2 \leq 4M^2 \\ \sqrt{-M - \frac{1}{2} + \sqrt{\left(\frac{\Omega}{2}\right)^2 + M + \frac{1}{4}}} & \text{if } \Omega^2 > 4M^2 \end{cases}
\tag{S4}
$$

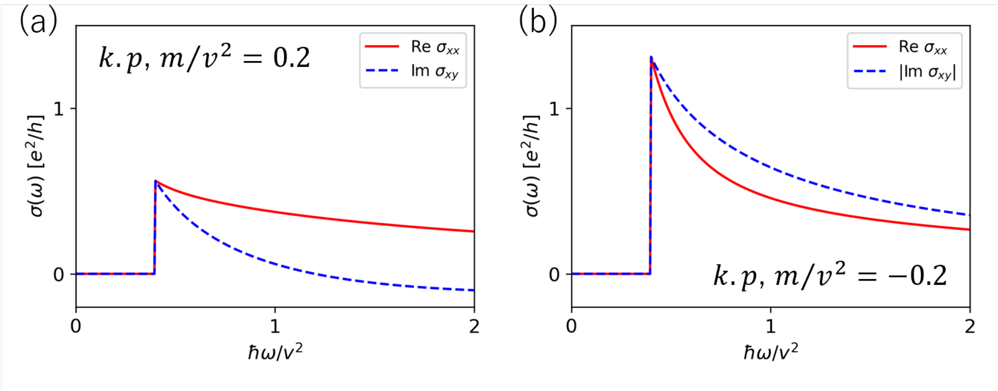

Figure S1: **Optical absorption in gapped Dirac systems.** The absorptive part of the optical conductivity, Re $\sigma_{xx}(\omega)$ and Im $\sigma_{xy}(\omega)$, are plotted for trivial phase ($m/v^2 = 0.2$, (a)) and topologically nontrivial phase ($m/v^2 = -0.2$, (b)). A larger optical response is obtained for the topological phase. At the optical absorption edge, $\hbar\omega = 2m$, $\approx$ 100% MCD, i.e., Re $\sigma_{xx}(\omega) \approx \left|\mathrm{Im}\ \sigma_{xy}(\omega)\right|$ is realized.

We calculated Re $\sigma_{xx}(\omega)$ and Im $\sigma_{xy}(\omega)$ analytically and the result for $m/v^2 = \pm 0.2$ is shown in Fig. S1. The optical response in the topological phase $m/v^2 = -0.2$ is more than twice as large as in the trivial phase $m/v^2 = 0.2$. This can be understood as the enhancement due to the non-trivial topology of the system, as explained in the main text. At the optical absorption edge, $\hbar\omega = 2m$, Re $\sigma_{xx}(\omega) = \left|\mathrm{Im}\ \sigma_{xy}(\omega)\right|$ holds and hence $\approx$ 100% MCD is realized around the optical absorption edge, as discussed in the main text.

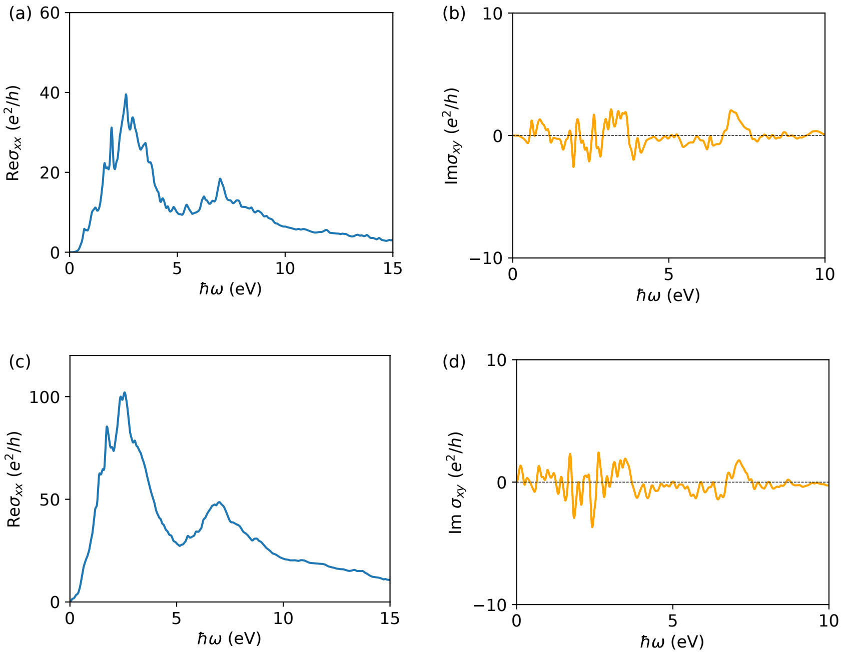

Figure S2: **Absorptive part of the optical conductivity over a wide photon energy window for 1SL and 3SL $MnBi_2Te_4$.** (a,b) The Re σ_{xx} for (a) 1SL, (b) 3SL. (c,d) The Im σ_{xy} for (c) 1SL (d) 3SL. The Re σ_{xx} is always positive and it steadily increases with the photon energy before reaching its peak value, while the Im σ_{xy} shows a bounded oscillatory pattern with the photon energy.

‘

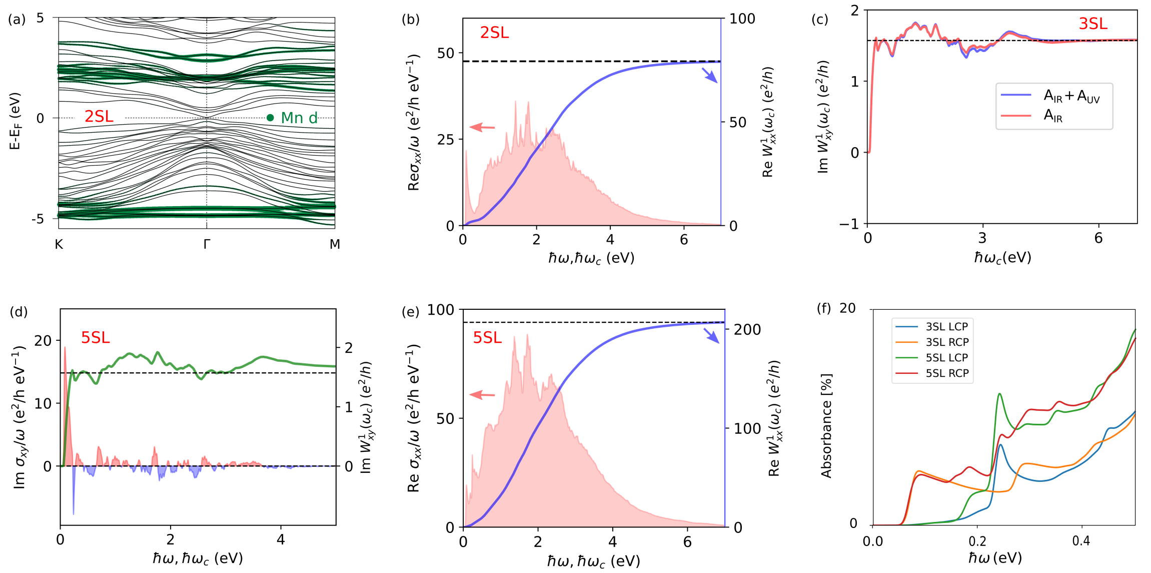

Figure S3: **Location of the Mn d bands and the different optical weights of thinfilm $MnBi_2Te_4$ of varying thickness.** (a) Orbital resolved band structure of 2SL $MnBi_2Te_4$. The Mn 3*d* bands are located away from the Fermi level for our choice of the onsite Coulomb potential, $U = 3$ eV. The low energy optical excitation of few SL $MnBi_2Te_4$ thus mostly involves the bands of Bi *p* and Te *p* orbital characters. (b) The Re σ_{xx}/ω (Re W^1_{xx}) over a wide photon energy (cutoff energy) window for 2SL $MnBi_2Te_4$. Although the $\mathcal{PT}$ symmetry forbids the optical Hall conductivity at all frequencies in even SL $MnBi_2Te_4$, the Re σ_{xx}/ω is non-zero, and it leads to a finite quantum weight. (c) Negligible effect of ignoring the UV part (A_{UV}) of the Berry connection on Im W_{xy}, in sharp contrast to the Re W_{xx} (see Fig.S7(f)). (d) The Im σ_{xy}/ω (Im W^1_{xy}) and (e) The Re σ_{xx}/ω (Re W^1_{xx}) for 5SL $MnBi_2Te_4$. The Chern insulator ground state of 5SL is evident. (f) Near-perfect MCD in the 3SL and 5SL $MnBi_2Te_4$ in the infrared region. In these plots, we only consider the optical transitions between the Mn *d*, Te *p*, and Bi *p* bands.

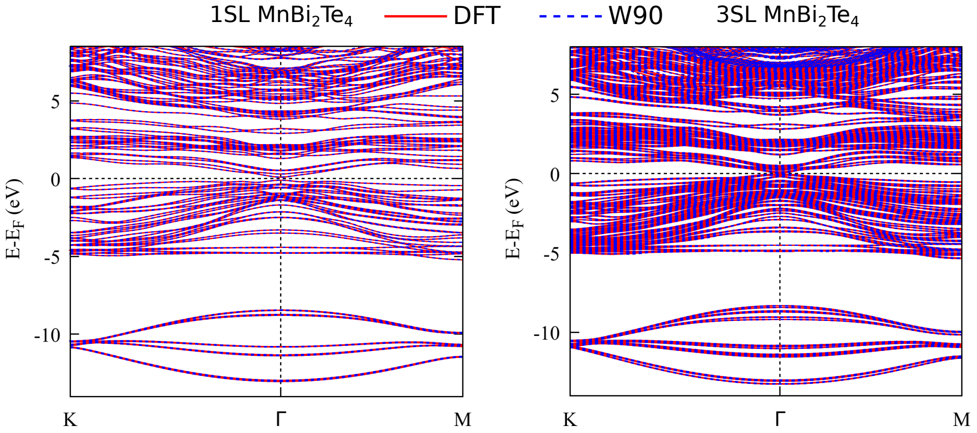

Figure S4: **Comparison of the DFT computed and Wannier interpolated (w90) band structure over a wide energy window demonstrating the high quality of the Wannier fitting.** (a) 1SL $MnBi_2Te_4$ and (b) 3SL $MnBi_2Te_4$. We have used a 'frozen-energy-window' of ~ -15 eV to ~ 8.5 eV for 1SL, and ~ -15 eV to ~ 11 eV (measured from the Fermi level) for the 3SL case during the disentanglement procedure. Such a wide energy window has been used only for correctly capturing the high frequency response.

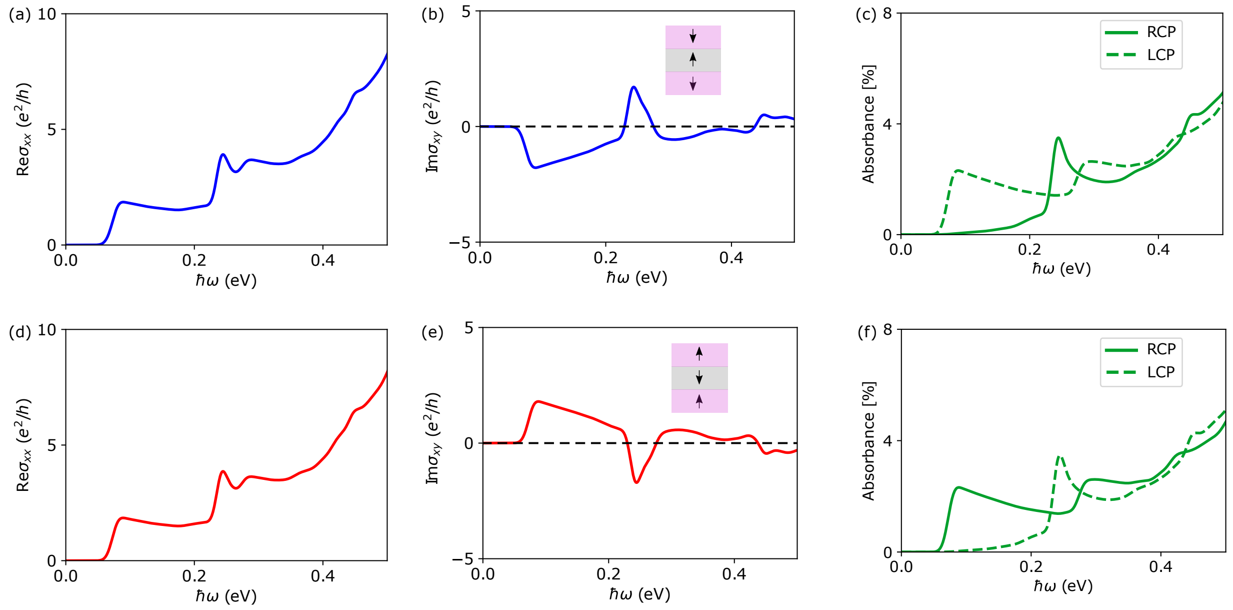

Figure S5: **Reversal of the handedness property of optical absorption on reversing the direction of global magnetization of 3SL $MnBi_2Te_4$.** Panels (a-c) show the ↑↓↑ configuration, while panels (d-f) display the ↓↑↓ configuration. The sign change of Im$\sigma_{xy}(\omega)$ upon changing global magnetization results in the complete absorption of circularly polarized light of opposite helicity in these two different magnetic configurations.

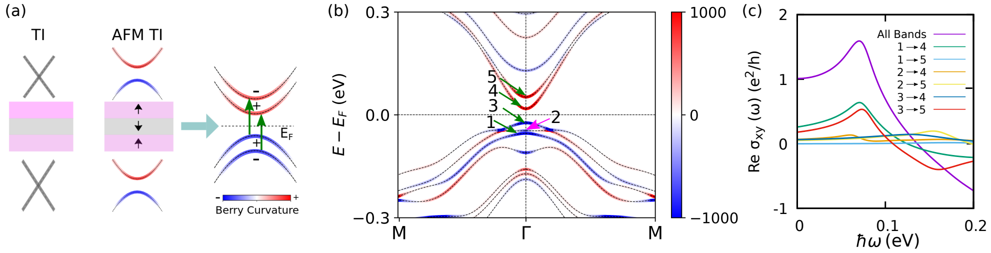

Figure S6: **Identification of the "gapped Dirac bands".** (a) A schematic illustration of the low energy gapped Dirac cone states of 3SL $MnBi_2Te_4$. (same as the Fig.2(d) of the main text) (b) The band and momentum resolved Berry curvature distribution in 3SL $MnBi_2Te_4$. The low energy bands near the Fermi level are denoted by numbers. The bands indicated by 1,3,4, and 5 are the ones that represent "gapped Dirac bands". (c) The band resolved contribution of Re$\sigma_{xy}(\omega)$ arising from different low-energy band pairs. Clearly, the largest contributions arise from 1 $\longrightarrow$ 4, and 3 $\longrightarrow$ 5 pairs of bands justifying the selection of the Dirac bands.

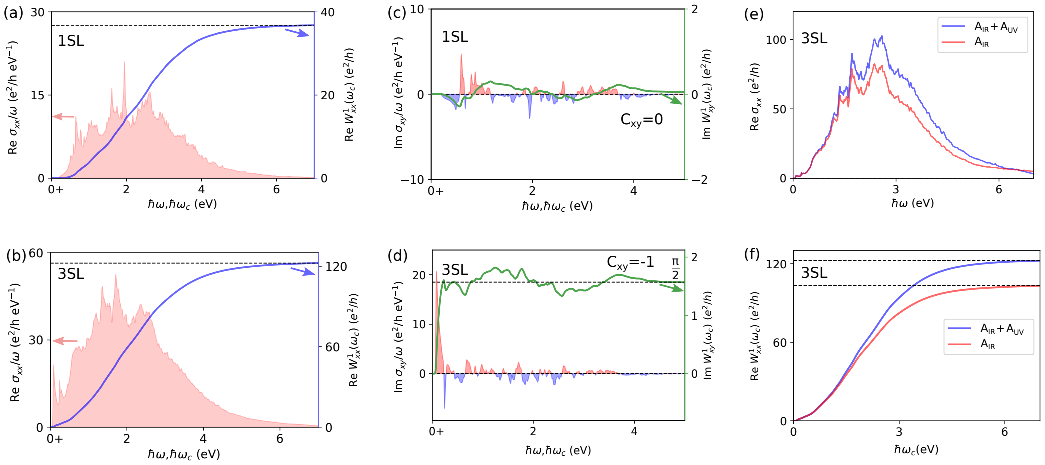

Figure S7: **Optical conductivity and optical weight of a few SL thick $MnBi_2Te_4$ in a wide photon energy window.** Unlike Fig.6 of the main text, here we only consider the optical transitions involving the Mn d, Te p, and Bi p orbital manifold. (a,b) The Re σ_{xx}/ω (Re W^1_{xx}) over a wide photon energy (cutoff energy) window for (a) 1SL and (b) 3SL $MnBi_2Te_4$. (c,d) The Im σ_{xy}/ω (Im W^1_{xy}) over a wide photon energy (cutoff energy) window for (c) 1SL and (d) 3SL $MnBi_2Te_4$. In the case of 1SL, the Im W^1_{xy} converges to zero, while for the 3SL, the Im W^1_{xy} converges to $e^2/4\hbar$, revealing their trivial and the non-trivial ground state, respectively. (e,f) Effect of ignoring the UV part ($\mathbf{A}^{UV}$) of the interband Berry connection on (e) Re σ_{xx}, and (f) Re W^1_{xx}. The larger spread of the Wannier orbitals in this case results in a significant contribution arising from the UV part.
